# Facial Neuralgia

**Published:** 1883-08

**Authors:** 


### Editorial.

### FACIAL NEURALGIA.

There are few disorders that so perplex and puzzle the dental practitioner as affections of the fifth pair of cranial nerves. He is so accustomed to visits from patients who complain of "neuralgia," but who, in by far the greater number of instances, are suffering from neglect of the teeth, that he is always very loth to admit that the pain is not due to some dental cause. Continued irritation of the terminal filaments of the maxillary branches of the trifacial nerves, due to decayed teeth, will often produce such a hyperæsthetic condition as very closely to simulate, if it does not really produce, an irritation of the nerve trunks. Again, irritation of a dental filament is frequently reflected along the course of the nerve until the pain is distinctly felt at a distance from the real location. This may even be upon the other jaw, and more rarely upon the opposite side of the face. Not infrequently dental irritation may manifest itself in the neighborhood of the supra or infra-orbital foramen, or in the temporal region. It is sometimes the cause of conjunctivitis and other affections of the eye. Any of the tissues, in fact, which are supplied by branches of the trifacial nerve, may be the apparent source of the pain which is wholly due to affections of the teeth.

It is not strange, then, that the dental or the general practitioner should at times find extreme difficulty in diagnosing such cases. It is only after the most careful examination by an entirely competent dentist, that any one should consider a resort to constitutional treatment, or to general surgical interference at all necessary. Even the most thorough search may after all fail to reveal a dental lesion, for there are pathological changes in the tooth pulp which it is at times impossible to diagnose. There are also hypertrophies, or exostoses of the roots of teeth, which are only revealed by extraction. Impaction, too, of unerupted teeth, may be the hidden source of the most intense pain, only felt at some distance from the real location. It may readily be seen, therefore, that general surgery is without doubt sometimes appealed to, and a section made of one or more of the branches of the Trigeminus, without any absolute necessity, and the wisdom of a consultation with the thoroughly qualified dentist before proceeding to extreme measures in any case is apparent. We might illustrate this by numerous instances in our own practice, but perhaps one will suffice.

Mrs. D., who was in the seventh month of pregnancy, had a long history of miscarriages. This was the eighth time, and she had never yet gone to full term, or brought into the world a living child. She was now in the hands of an eminent physician who was making the most strenuous efforts to carry her through. There had been from the fourth month a constant succession of various mishaps and illnesses, and the physician had waged a constant, unintermitting warfare, thus far successfully. She had passed the usual periods of her previous miscarriages, and her hopes as well as those of her medical attendant were high, that the time of danger was over, when she was seized with the most violent neuralgic pains, darting successively along all the three branches of the Trigeminus. Every remedy known to medical science was employed without permanent benefit, and her physician had begun seriously to contemplate the dread alternative of premature delivery, although it implied the complete failure of all his long-directed efforts, and the blasting of the hopes so fondly entertained.

At this point we were summoned by the physician, as a kind of last resort, but with little expectation that the source of the trouble would be found in the mouth, as she had a beautiful set of teeth of which she took extreme care, and had never required the services of the dentist. The patient was found bolstered up in bed, and undergoing the most excruciating pain. The first act was to procure a glass of hot water and request her to hold a liberal quantity in her mouth, and tell its effect. The pain was rather exacerbated by it, but as it produced a marked result we felt certain we were upon the right track. A mouthful of ice-water was then substituted, with the effect of producing a marked mitigation, and from these simple indications a diagnosis of pulp inflammation was at once made, which the result proved was correct. From a dental syringe alternate streams of hot and cold water were thrown upon single teeth successively, until the trouble was located in a superior first bicuspid. By means of a bit of floss silk passed between the teeth and sawed laterally back and forth, a large cavity was located high up beneath the gum, upon the distal surface. An enamel chisel was used to break through the tooth wall, and a sharp excavator then thrust through the thin septum of dentine directly into the pulp, with the result of an instant relief from all pain, by depleting the engorged tissues, which had previously been confined within the unyielding bony walls of the tooth. The proper dressings were applied, and the distressing "neuralgia" troubled her no more.

In this case the pain had never been located in the affected tooth, but had assumed all the appearances of genuine trifacial neuralgia, and only for the persistent skepticism which was the result of an extended experience, we should undoubtedly have pronounced it such.

But although we firmly believe that a large proportion of the so-called cases of neuralgia are directly tracable to dental disturbances, the surgeon will occasionally meet with instances which are due to other causes. We had such an one under our care recently. It was a pale, anæmic, chlorotic young girl, the most of whose lower teeth upon one side had been removed without relief from the pain which seemed to be located in them, and which immediately betook itself to another as soon as the apparently affected one was extracted. Electricity was used, and the following prescription ordered :

℞ Ferri et Quin. cit ʒ ii.

Syrup Aurantii, ℥ i.

Aqua Dist. ℥ i.

Elixir Calisaya, ℥ ii.

Sig. *Coch. Parv. ter in die.*

The pain vanished with the other general symptoms. Aside from dental or constitutional disturbances, there are lesions of the nerve itself, which sometimes demand surgical interference for relief from the terrible and persistent agony. We publish on another page two very interesting cases in the practice of Dr. S. Weir Mitchell, of Philadelphia, in one of which novel means were used to prevent the re-establishment of the diseased nerve. A letter from Dr. Mitchell informs us that the cement used to close the foramen was a preparation of gutta-percha. It is intended to leave it in position permanently, unless it shall cause inflammatory trouble. The final result of the operation will be looked for with interest. Up to the present time it seems successful.

A careful examination will usually enable the skillful dentist to distinguish between neural pains, the result of dental irritation, and actual nerve lesions. If it be the former, some tooth will ordinarily be found tender upon sharp percussion, or peculiarly sensitive to thermal changes. If the cause be an impacted or non-erupted tooth, there is usually local inflammation, perhaps muscular soreness, and difficulty in mastication, or swallowing. If there be pathological changes within the tooth pulp, there will sometimes be a sensation of fullness and pressure in the tooth, with a gnawing, uneasy feeling, quite distinct from the darting or shooting pains along the course of a really affected nerve.

Hypertrophy of cementum, or exostosis, is perhaps the most difficult of diagnosis of any of the dental diseases which simulate trifacial neuralgia. But even in this closely marked and obscure disease the careful observer will frequently detect something which may serve to give him an insight into the source of trouble. The body of the bone may present a protuberance, more or less marked, or the tooth will seem abnormally firm in its socket, and sore at times when used in mastication.

When none of these symptoms are discernible upon the most careful inspection, we may begin to suspect undoubted neuralgia, and should proceed to a more critical examination of the character and location of the pain, and endeavor to trace the course of the darting paroxysms. If the pains be unilateral, are sudden in their attack, and of a shooting, boring, or burning character, if they are markedly intermittent, and if they leave the parts chiefly affected sore and tender to the touch, and especially if they appear to follow the course of a particular nerve, we may begin to be somewhat sure of their character. The constitutional condition of the patient is a great help too, in making out the diagnosis. If there is a state of debility, nerve lesions may be suspected. If the affection is of considerable standing, sore spots may be detected wherever the general course of the nerve is changed, and about the foramen of exit from the bone. The most common locations for these are about the supra and infra-orbital, and the mental foramen, and in the temporal regions. Finally, one grand distinguishing characteristic of all neuralgias is that great bodily or mental fatigue and all depressing and dispiriting influences predispose to an attack, and aggravate it when existing. There are other symptoms which may be relied upon at times, but these are the leading ones, and when these are found concurrent, an approximately clear diagnosis may be made.

The successful treatment of trifacial neuralgia is at times extremely difficult. First, the general health should be carefully looked to, and the tone of the system restored if it be lowered, by generous diet, tonics, and entire rest and relief from all disturbing influences. Quinine may be advantageously prescribed before the attacks, and in some instances small doses of strychnine are beneficial. Electricity is useful when the trouble is in the nerve centres, but it is apt to be injurious when the disease arises from excitement of the nerves themselves, or from irritation of their terminal filaments. Subcutaneous injections of morphia usually give temporary relief from the paroxysms. Dr. A.E. Cartledge, in *The Medical and Surgical Reporter*, recommends ether spray, and we have used it with marked beneficial effect, when the pain was accompanied or succeeded by sore spots. The eye or the ear may be protected by pieces of oiled silk or rubber dam, when, by means of the foot bellows and the Richardson spray apparatus, the temperature of the tender points may be reduced until all sensation is entirely suspended. The operation should be kept up for eight or ten minutes, and this will usually be followed by marked diminution, or perhaps entire suspension of the paroxysms.

Deeper lesions of the nerves may require operations which only professional surgeons will be willing to undertake, but these are to be resorted to only when all other measures fail.

